# Supplementary material for: Secondary Resistant Mutations to Small Molecule Inhibitors in Cancer Cells
Source: Cancers (Basel). 2020 Apr 9;12(4):927. doi: 10.3390/cancers12040927 (PMC7226513; doi:10.3390/cancers12040927)
Supplement: Supplementary file 1 [file cancers-12-00927-s001.pdf]

Supplementary Material: Secondary Resistant Mutations to Small Molecule Inhibitors in Cancer Cells

Abdulaziz B. Hamid and Ruben C. Petreaca *

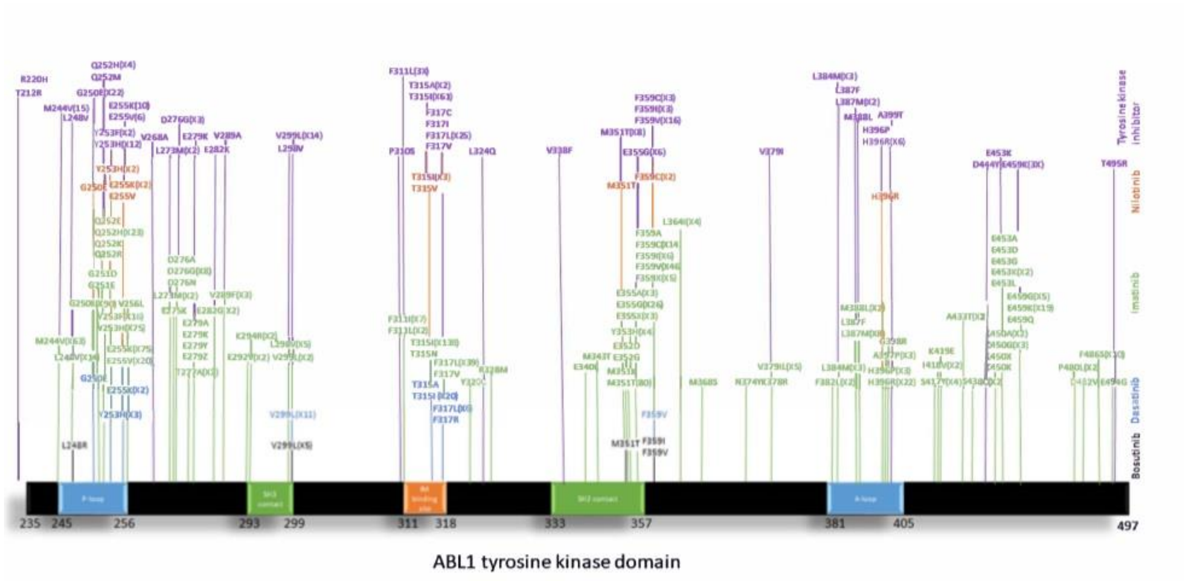

Supplementary Figure S1. Resistant mutations in the ABL1 tyrosine kinase domain.

The following tables list the resistant mutations and references deposited on COSMIC [1] and listed in the file "COSMIC Resistant Mutations" (https://cancer.sanger.ac.uk/cosmic/download). The file provides PubMed ID for every mutation. We searched the provided PubMed IDs and listed these references.

Supplementary Table S1. Resistant mutations in non-small cell lung cancer (NSCLC).

Gene name	Drug name	Mutation	References
ALK	Alectinib	p.V112L, p.V1180L, p.V803L	[2]
		p.I103N/S, p.I1171N/S, p.I794N/S	[3]
		p.I103N, p.I1171N, p.I794N	[4]
		p.I1171S	[5]
		p.G1202R, p.G134R, p.G825R	[6]
	Ceritinib	p.F1174C/V, p.G1202R, p.G1269A, p.L1196M	[7]
		p.G1123S, p.G55S, p.G746S	[8]
		p.D1203N, p.D135N, p.D826N	[9]
	Crizotinib	p.F106L, p.F1174L, p.F797L	[10]
		p.G1202R, p.G134R, p.G825R, p.L1196M, p.L128M, p.L819M, p.S1206Y, p.S138Y, p.S829Y, p.T1151dup, p.T774dup, p.T83dup	[11]
		p.G1269A, p.G201A, p.G892A, p.L1196M, p.L128M, p.L819M	[12]
		p.C1156Y, p.C779Y, p.C88Y, p.G1269A, p.G201A, p.G892A, p.L1196M, p.L128M, p.L819M	[13]
		p.G1269A, p.S1206Y	[7]
		p.F106V, p.F1174V, p.F797V, p.G1202R, p.G134R, p.G825R	[14]
		p.I103T, p.I1171T, p.I794T	[4]
		p.C1156Y, p.C779Y, p.C88Y, p.G1269A, p.G201A, p.G892A	[15]
		p.G1128A, p.G60A, p.G751A	[16]
EGFR	Afatinib	p.T790M	[17–20]

		p.T745M, p.T790M, c.*359C > T	[21]
	Erlotinib	p.T790M	[22–35]
		p.T745M, p.T790M, c.*359C > T	[36–42]
	Gefitinib	p.T790M	[23,27,33,43–80]
		p.T745M, p.T790M, c.*359C > T	[38,81–97]
		p.D716Y, p.D761Y, p.T790M, c.*271G > T	[22]
	HM61713	p.C752S, p.C797S, c.*380G > C	[98]
	Osimertinib	p.C797S	[99]
		p.L673Q, p.L718Q, c.*143T > A	[97]
		p.G796D	[79]
		p.C752G, p.C752S, p.C797G/S, p.G751R/S, p.G796R/S, p.L747F/H, p.L792F/H, c.*364C>T, c.*365T>A, c.*376G>A/C, c.*379T > A/G, c.*380G > C	[42]
		p.L702P, p.L747P, c.*229_*230delinsCC	[100]
	Not Specified TK Inhibitor	p.T790M	[99, 101–106]
		p.T745M, p.T790M, c.*359C > T	[38]
	XL647	p.T790M	[107]
MET	Afatinib	unknown	[108]
	Capmatinib	p.D1228N, p.D1246N, p.Y1230H, p.Y1248H	[78]
	Crizotinib	p.D1228N, p.D1246N	[109]
		p.Y1230C, p.Y1248C	[110]
		p.D1246H/N, p.Y1248H	[111]
		p.D1228N, p.D1246N, p.Y1230H, p.Y1248H	[78]
		p.D1246N, p.G1181R, p.Y1248H/S	[112]
		p.D1246H/N, p.D1249Y, p.Y1248H	[80]
	Erlotinib	unknown	[113]
	Gefitinib	unknown	[78]
	Osimertinib	unknown	[35,80]
	Savolitinib	p.D1228V, p.D1246V	[108]
KIT	Crizotinib	p.D816G	[114]
NF2	Afatinib	p.R115*, p.R156*, p.R157*, p.R198*, c.447 + 13384C > T, c.447 + 22703A > T	[21]

Supplementary Table S2. Resistant mutations detected in cancers of the hematopoietic and lymphoid tissues.

Gene name	Drug Name	Mutation	Reference
ABL1	Bosutinib	p.F359V, p.M351T	[115]
		p.F359I, p.F378I, p.L248R, p.L267R	[116]
		p.V299L	[117]
	Dasatinib	p.F317L	[118]
		p.E255K, p.F317L	[119]
		p.F317L, p.G250E, p.T315I, p.Y253H	[120]
		p.F317L, p.T315I, p.Y253H	[115]
		p.E255K, p.T315I	[121]
		p.F317L, p.T315I	[122]
		p.F317R, p.F336R, p.T315A	[116]
		p.V299L	[117]
		p.F359V, p.T315I, p.V299L	[123]
	Imatinib	p.E255K/V, p.E274K/V, p.H396P, p.H415P, p.T315I, p.T334I, p.Y253H, p.Y272H	[123]
		p.E255K, p.E274K, p.F317L, p.F336L, p.G250E, p.G269E, p.M351T, p.M370T, p.T315I, p.T334I, p.Y253H, p.Y272H	[124]
		p.E255K, p.E355G, p.F317L, p.F359V, p.F382L, p.G250E, p.H396R, p.L387M, p.M244V, p.M343T, p.M351T, p.Q252H/R, p.T315I, p.V379I, p.Y253F/H	[125]

	p.E255K/V, p.E274K/V, p.E355G, p.E374G, p.H396R, p.H415R, p.M244V, p.M263V, p.M351T, p.M370T, p.Q252H, p.Q271H, p.T315I, p.T334I, p.Y253F/H, p.Y272F/H	[126]
	p.E255K/V, p.E274K/V, p.E355G, p.E374G, p.E459K, p.E478K, p.F317L, p.F336L, p.F359V, p.F378V, p.F486S, p.F505S, p.G250E, p.G269E, p.H396R, p.H415R, p.L248V, p.L267V, p.M244V, p.M263V, p.M351T, p.M37.;/6y7uuuuuu650T, p.Q252H, p.Q271H, p.S417Y, p.S436Y, p.T315I, p.T334I, p.Y253F, p.Y272F	[127]
	p.D276G, p.D295G, p.E255K/V, p.E274K/V, p.F317L, p.F359A/V, p.F378V, p.H396R, p.H415R, p.M351T,, p.M370T, p.T315I/N, p.T334I/N, p.Y253H, p.Y272H	[128]
	p.M351T, p.Q252E, p.Y253H	[129]
	p.L248V, p.L267V	[130]
	p.M244V	[118]
	p.A433T, p.D276G, p.E255K/V, p.E292V, p.E355A/G, p.E453K, p.E459G/K/Q, p.F311I, p.F317L, p.F359C/V, p.F486S, p.G250E, p.H396R, p.L248V, p.L298V, p.L364I, p.L387M, p.M244V, p.M351T, p.M388L, p.Q252H, p.T315I, p.Y253F/H	[131]
	p.D482V, p.E255K, p.E279Z, p.E282G, p.E292V, p.E352D/G, p.E355G, p.E450G, p.E453D/K, p.E459G, p.F359I/V, p.F382L, p.F486S, p.G250E, p.G251D, p.H396P/R, p.I418V, p.L248V, p.L273M, p.L364I, p.L387M, p.M244V, p.M351T, p.M388L, p.Q252H, p.R328M, p.S417Y, p.T315I, p.V379I, p.Y253F/H	[132]
	p.E255K/V, p.E450K, p.E459K, p.F317L, p.F359C/V, p.G250E, p.H396R, p.M244V, p.M351T, p.P480L, p.S417Y, p.T315I, p.Y253H	[133]
	p.D276G, p.E255K, p.E355G, p.F311I, p.F317L, p.G250E, p.H396R, p.L248V, p.L273M, p.L384M, p.M244V, p.T315I, p.Y253F/H	[134]
	p.E355G, p.F359V, p.G250E, p.H396R, p.L248V, p.M244V, p.N374Y, p.T315I, p.Y253F/H	[135]
	p.F378V, p.Y272H, p.M263V, p.G269E, p.L406M, p.Q271H, p.Y272F, p.F336V, p.L267V, p.F336L, p.T334I, p.Y253H, p.F317L, p.F359V, p.Y253F, p.F317V, p.M244V, p.G250E, p.Q252H, p.L364I, p.L248V, p.L387M, p.T315I	[136]
	p.E255K, p.E450A/G, p.E459K, p.F317L, p.G250E, p.T315I, p.T334I, p.Y253H	[137]
	p.G250E	[138]
	p.T315I, p.G250E, p.M244V, p.A397P, p.Y253H, p.F359X, p.D276G, p.L248V, p.M351T, p.E355X, p.L384M, p.E255K, p.F359V, p.F317L, p.H396R, p.E279Y, p.E450X/A, p.E453L, p.E459K, p.L298V, p.E355G, p.I418V, p.F359C	[139]
	p.A433T, p.E255K, p.E459K, p.F317L, p.G250E, p.M244V, p.Q252H/K, p.T315I, p.Y253H	[119]
	p.A397P, p.E255K/V, p.E355G, p.F311L, p.F317L, p.F359I/V, p.F486S, p.G250E, p.H396R, p.L248V, p.L298V, p.L364I, p.M244V, p.M351T, p.Q252H, p.Y253H	[140]
	p.E255K/V, p.E459K, p.F311I, p.F359V, p.F486S, p.G250E, p.M244V, p.Q252H, p.T315I, p.Y253F/H,	[141]
	p.F311I, p.F317L, p.G250E, p.K294delinsRGG, p.K313delinsRGG, p.Y253H	[120]
	p.E255K/V, p.E459K, p.F317L, p.F359V, p.G250E, p.M351T, p.T315I, p.Y253H	[115]
	p.E255K/V, p.E355G, p.F311I, p.F359C, p.F359I, p.F359V, p.G250E, p.L248V, p.L298V, p.M244V, p.M351T, p.Q252H, p.T315I, p.V289F, p.Y353H	[142]
	p.H396P, p.H415P, p.M244V, p.M263V	[143]
	p.Q252H, p.T315I	[144]
	p.E255K, p.E279K, p.E355G, p.E459K, p.F359C, p.F486S, p.G250E, p.H396R, p.K419E, p.L387M, p.M244V, p.M351T, p.Q252H, p.T315I, p.Y253H	[122]
	p.F359I, p.F378I	[116]
	p.A399T, p.E255K/V, p.E355G, p.E450G, p.E494G, p.F317L, p.F359I/V, p.F486S, p.G250E, p.H396R, p.K378R, p.M244V, p.M351K/T, p.M351T, p.S438C, p.T277A, p.T315I, p.V256L, p.V299L, p.Y253H, p.Y320C	[145]
	p.V299L	[117]
	p.E255K, p.M351T, p.M370T, p.T315I, p.T334I, p.V289F, p.Y253H	[146]
	p.D276A, p.F311L, p.F317L, p.F359V, p.G250E, p.L340L, p.M244V, p.M351T, p.S417Y, p.T277A, p.T315I, p.V379I, p.Y253H	[147]

		p.D276N, p.D295N, p.E255K/V, p.E274K/V, p.E279A, p.E298A, p.E355G, p.E374G, p.E453A, p.E459G, p.E472A, p.E478G, p.F317L, p.F336L, p.F359C/V, p.F378C/V, p.G250E, p.G269E, p.H396R, p.H415R, p.L387F, p.L387M, p.L406F/M, p.M244V, p.M263V, p.M351T, p.M370T, p.S438C, p.S457C, p.T315I, p.T334I, p.Y253H, p.Y272H	[148]
		p.E255K, p.E275K, p.E355G, p.E453G, p.E459K, p.F317L, p.F359V, p.G250E, p.M351T, p.Q252H, p.Y253F/H	[149]
		p.A397P, p.D276G, p.E255K, p.E355A, p.E355G, p.F359C, p.G250E, p.G251E, p.H396R, p.L387M, p.M351T, p.N368S, p.T315I, p.V289F, p.Y253H	[150]
		p.E355G, p.G398R	[151]
		p.E255K, p.E274K, p.G250E, p.G269E	[152]
		p.G250E, p.L384M, p.V379I, p.Y253H, p.Y272H	[153]
	Nilotinib	p.M351T	[118]
		p.E255K/V, p.F359C	[119]
		p.G250E	[115]
		p.T315I	[144]
		p.H396R, p.Y253H	[122]
		p.T315V, p.T334V	[116]
	Not specified TK inhibitor	p.E355G, p.F317L, p.F359I, p.G250E, p.T495R, p.V299L	[154]
		p.D444Y, p.E255K, p.E355G, p.F317L, p.G250E, p.L273M, p.T315I, p.V299L, p.V379I, p.Y253F/H, p.Y353H	[155]
		p.D276G, p.D295G, p.F317L, p.F336L, p.F359V, p.F378V, p.G250E, p.G269E, p.L387M, p.L406M, p.M244V, p.M263V, p.M388L, p.M407L, p.T315I, p.T334I, p.Y253H, p.Y272H	[156]
		p.A399T, p.E255V, p.E282K, p.F311L, p.F317L, p.F359I, p.G250E, p.H396R, p.L384M, p.M244V, p.Q252H, p.R220H, p.T315I, p.V289A, p.V299L	[157]
		p.F317L, p.G250E, p.G269E, p.T315I, p.T334I, p.V299L	[158]
		p.D276G, p.E279K, p.F317L, p.F359C/I/V, p.G250E, p.H396R, p.L273M, p.L387F, p.M244V, p.M351T, p.Q252H, p.T212R, p.T315I	[159]
		p.D325G, p.E255K/V, p.E355G, p.E459K, p.F311L, p.F317C, p.F317I/L/V, p.F359C/V, p.G250E, p.H396R, p.L248V, p.L298V, p.L384M, p.L387M, p.M244V, p.M351T, p.Q252H, p.T315A/I, p.V268A, p.V299L, p.V338F, p.Y253H	[160]
BTK	Ibrutinib	p.C481S, p.C515S, c.1039-1483T>A	[161]
		p.C481F/S, p.C515F/Sc.1039-1482G>C/T	[162]
		c.1039-1482_1039-1481delinsCT, c.1039-1482G>C	[163]
		p.C481F/R/S/Y	[164]
		p.C481S, p.C515S, c.1039-1482G>C	[165]
		p.T316A, p.T350A	[166]
		p.C481R/S, p.C515R/S, c.1039-1482G>C, c.1039-1483T>A/C	[167]
FLT3	Quizartinib	p.D835Y	[168]
		p.D835F/V/Y, p.F691L	[169]
		p.F691L	[170]
	Sorafenib	p.D835H/X/Y	[171]
		p.D835H, p.F691L	[172]
	Sunitinib	p.D835Y	[172]

Supplemental Table S3. Resistant mutations in gastrointestinal stromal tumors (GIST) soft tissue.

Gene Name	Drug Name	Mutation	References
ALK	Crizotinib	p.F106L, p.F1174L, p.F797L	[10]
BRAF	Imatinib	p.V600E, p.V640E	[173]
KIT	Imatinib	p.D716N, p.D816G, p.D820E/Y, p.N822K, p.T670I, p.V654A	[174]
		p.D820Y, p.N822K, p.T670I, p.V654A, p.Y823D	[175]
		p.D816E, p.D820E/G/Y, p.N822K, p.S709F, p.T670E/I, p.V654A, p.Y823D	[176]
		p.C809G, p.D816H, p.D820A/E/G, p.N822K/Y, p.T670I, p.V654A, p.Y823D	[177]

		p.C809G, p.D816H, p.N822K, p.V654A, p.Y823D	[178]
		p.C809G, p.D816E, p.D820E/G/Y	[179]
		p.V654A	[180]
		p.A829P, p.D816H, p.D820G/Y, p.N822K/Y, p.T670I, p.V654A, p.Y823D	[181]
		p.V654A	[182]
		p.A829P, p.C809G, p.D816A/H, p.D820A/G/Y, p.N822K, p.T670I, p.V654A, p.Y823D	[183]
		p.T670I	[184]
		p.D816H	[185]
		p.K642E, p.Y823D	[186]
		p.V654A	[187]
		p.A829P, p.D820Y, p.N822K, p.V654A, p.Y823D	[188]
		p.V654A	[173]
		p.S821F	[189]
		p.D579del, p.D820E/G/Y, p.K642E, p.K818_D820 > N, p.N680K, p.N822K/Y, p.T670E/I, p.V569_Y578del, p.V654A, p.Y578C, p.Y823D	[190]
		p.N822K	[191]
		p.A829P, p.V654A	[192]
		p.D820V/Y, p.V654A, p.Y823D	[193]
	Nilotinib	p.N655T	[194]
	Sunitinib	p.N822K	[195]
MAP2K1	PD0325901	p.F129L	[196]
MAP2K2	PD0325901	p.V215E	[196]
PDGFRA	Imatinib	p.D842V	[174,175,177,183,186,197–201]
		p.D842V, p.D842_D846delinsG, p.I843_S847delinsT	[202]
PDGFRA	Sunitinib	p.D842V	[189]
AC058822.1	Imatinib	p.D602V	[198]

Supplementary Table S4. Resistant mutations in melanoma.

BRAF	Dabrafenib	c.139_1140del, c.139-?_1314+?del, c.505-?_1140+?del	[203]
		c.139-?_1314+?del	[204]
	Vemurafenib	c.139_1140del, c.505-?_1140+?del	[203]
		p.L505H, p.L545H, c.139_1140del	[205]
		c.139-?_1314+?del, c.981-?_?del?	[206]
		p.V47_D380del	[207]
CTNNB1	Imatinib	p.S26C, p.S33C	[208]
JAK1	Pembrolizumab	p.Q503*	[209]
JAK2	Pembrolizumab	c.1641+2T > G	[209]
MAP2K1	Vemurafenib	p.E203K, p.Q56P	[210]
MAP2K2	Dabrafenib	p.C125S, p.C28S, p.E110K, p.E207K	[211]
		p.Q60P	[204]
	Vemurafenib	p.F57C, c.-122T > G	[203]
NRAS	Dabrafenib	p.Q61K	[212]
		p.G12D, p.Q61K	[211]
	Vemurafenib	p.Q61K/R	[206,213]
PIK3CA	Vemurafenib	p.E545K	[205]
PTEN	Vemurafenib	p.R159S	[204]
SMO	Vismodegib	p.V321M, p.W281L	[214]
		p.D473Y, p.G497W	[215]
		p.A459V, p.C469Y, p.T241M, p.V321M	[216]

		p.D473G/H/N, p.F460L, p.H231R, p.Q477E, p.S533N, p.V321A, p.W281C, p.W535L/R	[217]

Supplementary Table S5. Resistant mutations in breast cancer.

Gene name	Drug name	Mutation	References
ESR1	Endocrine therapy	p.Y276S, p.Y537S, c.*485A>C, c.851-26478A>C	[218]
		p.D277G, p.D538G, p.L275Q, p.L536Q, p.Y276S, p.Y537S, c.*482_*483delinsAG, c.*485A > C, c.*488A > G, c.851-26475A > G, c.851-26478A > C, c.851-26481_851-26480delinsAG	[219]
		p.D538G, p.Y537S, p.Y537N/C, p.D277G, p.Y276N/C/S, c.*484T > A, c.*485A > C, c.*485A > G, c.*488A > G, c.851-26475A > G, c.851-26478A > C/G, c.851-26479T > A	[220]
		p.D277G, p.D538G, c.*488A>G, c.851-26475A>G	[221]
		p.D538G, p.Y537S	[222]
		p.D538G, p.Y537C/N/S	[223]
		p.D538G, p.L536_D538>P, p.Y537C/N/S	[224]
		p.Y276N, p.Y537N, c.*484T > A, c.851-26479T > A	[225]
		p.D277G, p.D538G, p.L275H, p.L536H, p.Y276C/N/S, p.Y537C/N/S, c.*482T > A, c.*484T > A, c.*485A > C/G, c.*488A > G, c.851-26475A>G, c.851-26478A > C/G, c.851-26479T > A, c.851-26481T > A	[226]
MAP2K1	PD0325901	p.L115P	[196]
MTOR	Rapamycin	p.F2108L	[227]

Supplementary Table S6. Androgen receptor (AR) resistant mutations in prostate cancer.

Drug name	Mutation	References
Abiraterone	p.H733R, p.T346A, p.T695A, p.T878A	[228]
	p.H733Q/R, p.T346A, p.T346S, p.T695A, p.T695S, p.T878A/S	[229]
Androgen	p.V184M, p.V526M, p.V716M	[230]
	p.H733R, p.T346A, p.T695A, p.T878A	[228]
	p.H733R, p.T346A, p.T695A, p.T878A	[231]
Enzalutamide	p.F345L, p.F694L, p.F877L, p.H733R, p.T346A, p.T695A, p.T878A, p.V732A	[231]
	p.T878A	[232]
Flutamide	p.H733Q, p.T346S, p.T695S, p.T878S	[233]
	p.H733R, p.T346A, p.T695A, p.T878A	[234]
	p.H733R, p.Q109*, p.Q451*, p.Q641*, p.T346A, p.T695A, p.T878A	[235]
	p.V184M, p.V526M, p.V716M	[236]
Ketoconazole	p.H733R, p.T346A, p.T695A, p.T878A	[228]
LHRH	p.H733R, p.T346A, p.T695A, p.T878A	[237]

Supplementary Table S7. Resistant mutations detected in other tissues.

Gene Name	Drug Name	AA Mutation	Primary Tissue	References
MET	PF-04217903	Not specified	kidney	[238]
MTOR	Everolimus	p.F313L, p.F2108L	thyroid	[239]
SMO	Vismodegib	p.D473H	central nervous system	[240]
BRAF	Dabrafenib	c.139-?_1314+?del	NS	[241]
MAP2K1	Vemurafenib	p.C121S	NS	[242]
		p.G128V, p.P124L, p.V60E	NS	[243]
MAP2K2	Dabrafenib	p.L46F, p.N126D, p.N29D, c.-156C>T	NS	[243]
	Vemurafenib	p.C125S, p.C28S, p.V35M, c.-189G>A	NS	[243]
NRAS	Dabrafenib Pembrolizumab	p.Q61/HK/R	NS	[243–245]

Vemurafenib

References

1. Tate, J.G., et al., *COSMIC: the Catalogue Of Somatic Mutations In Cancer.* Nucleic Acids Res, 2019. **47**(D1): p. D941-D947.
2. Katayama, R., et al., *Two novel ALK mutations mediate acquired resistance to the next-generation ALK inhibitor alectinib.* Clinical cancer research : an official journal of the American Association for Cancer Research, 2014. **20**(22): p. 5686-96.
3. Ou, S.-H.I., et al., *Identification of a novel HIP1-ALK fusion variant in Non-Small-Cell Lung Cancer (NSCLC) and discovery of ALK I1171 (I1171N/S) mutations in two ALK-rearranged NSCLC patients with resistance to Alectinib.* Journal of thoracic oncology : official publication of the International Association for the Study of Lung Cancer, 2014. **9**(12): p. 1821-5.
4. Toyokawa, G., et al., *Secondary mutations at I1171 in the ALK gene confer resistance to both Crizotinib and Alectinib.* Journal of thoracic oncology : official publication of the International Association for the Study of Lung Cancer, 2014. **9**(12): p. e86-7.
5. Ou, S.-H., et al., *ALK F1174V mutation confers sensitivity while ALK I1171 mutation confers resistance to alectinib. The importance of serial biopsy post progression.* Lung cancer (Amsterdam, Netherlands), 2016. **91**: p. 70-2.
6. Lin, Y.-T., et al., *Anaplastic Lymphoma Kinase (ALK) Kinase Domain Mutation Following ALK Inhibitor(s) Failure in Advanced ALK Positive Non-Small-Cell Lung Cancer: Analysis and Literature Review.* Clinical lung cancer, 2016. **17**(5): p. e77-e94.
7. Friboulet, L., et al., *The ALK inhibitor ceritinib overcomes crizotinib resistance in non-small cell lung cancer.* Cancer discovery, 2014. **4**(6): p. 662-673.
8. Toyokawa, G., et al., *Identification of a Novel ALK G1123S Mutation in a Patient with ALK-rearranged Non-small-cell Lung Cancer Exhibiting Resistance to Ceritinib.* Journal of thoracic oncology : official publication of the International Association for the Study of Lung Cancer, 2015. **10**(7): p. e55-7.
9. Wang, H.-Y., C.-C. Ho, and J.-Y. Shih, *Multiple Acquired Resistance Mutations of the ALK Tyrosine Kinase Domainafter Sequential Use of ALK Inhibitors.* Journal of thoracic oncology : official publication of the International Association for the Study of Lung Cancer, 2017. **12**(5): p. e49-e51.
10. Sasaki, T., et al., *The neuroblastoma-associated F1174L ALK mutation causes resistance to an ALK kinase inhibitor in ALK-translocated cancers.* Cancer Res, 2010. **70**(24): p. 10038-43.
11. Katayama, R., et al., *Mechanisms of acquired crizotinib resistance in ALK-rearranged lung Cancers.* Science translational medicine, 2012. **4**(120): p. 120ra17.
12. Kim, S., et al., *Heterogeneity of genetic changes associated with acquired crizotinib resistance in ALK-rearranged lung cancer.* J Thorac Oncol, 2013. **8**(4): p. 415-22.
13. Huang, D., et al., *Multiplexed deep sequencing analysis of ALK kinase domain identifies resistance mutations in relapsed patients following crizotinib treatment.* Genomics, 2013. **102**(3): p. 157-62.
14. Ignatius Ou, S.-H., et al., *Next-generation sequencing reveals a Novel NSCLC ALK F1174V mutation and confirms ALK G1202R mutation confers high-level resistance to alectinib (CH5424802/RO5424802) in ALK-rearranged NSCLC patients who progressed on crizotinib.* Journal of thoracic oncology : official publication of the International Association for the Study of Lung Cancer, 2014. **9**(4): p. 549-53.
15. Saber, A., et al., *Genomic Aberrations in Crizotinib Resistant Lung Adenocarcinoma Samples Identified by Transcriptome Sequencing.* PloS one, 2016. **11**(4): p. e0153065.
16. Ai, X., et al., *Next generation sequencing reveals a novel ALK G1128A mutation resistant to crizotinib in an ALK-Rearranged NSCLC patient.* Lung cancer (Amsterdam, Netherlands), 2018. **123**: p. 83-86.
17. Murakami, H., et al., *Phase I study of continuous afatinib (BIBW 2992) in patients with advanced non-small cell lung cancer after prior chemotherapy/erlotinib/gefitinib (LUX-Lung 4).* Cancer Chemother Pharmacol, 2012. **69**(4): p. 891-9.
18. Kim, Y., et al., *The EGFR T790M mutation in acquired resistance to an irreversible second-generation EGFR inhibitor.* Mol Cancer Ther, 2012. **11**(3): p. 784-91.
19. Wu, S.G., et al., *The mechanism of acquired resistance to irreversible EGFR tyrosine kinase inhibitor-afatinib in lung adenocarcinoma patients.* Oncotarget, 2016. **7**(11): p. 12404-13.
20. Campo, M., et al., *Acquired Resistance to First-Line Afatinib and the Challenges of Prearranged Progression Biopsies.* J Thorac Oncol, 2016. **11**(11): p. 2022-2026.

21. Pirazzoli, V., et al., *Acquired resistance of EGFR-mutant lung adenocarcinomas to afatinib plus cetuximab is associated with activation of mTORC1.* Cell Rep, 2014. **7**(4): p. 999-1008.
22. Balak, M.N., et al., *Novel D761Y and common secondary T790M mutations in epidermal growth factor receptor-mutant lung adenocarcinomas with acquired resistance to kinase inhibitors.* Clin Cancer Res, 2006. **12**(21): p. 6494-501.
23. Sequist, L.V., et al., *Genotypic and histological evolution of lung cancers acquiring resistance to EGFR inhibitors.* Sci Transl Med, 2011. **3**(75): p. 75ra26.
24. Querings, S., et al., *Benchmarking of mutation diagnostics in clinical lung cancer specimens.* PLoS One, 2011. **6**(5): p. e19601.
25. Lund-Iversen, M., et al., *Clinicopathological characteristics of 11 NSCLC patients with EGFR-exon 20 mutations.* J Thorac Oncol, 2012. **7**(9): p. 1471-3.
26. Sakai, A., K. Kasahara, and T. Sone, *Detection of EGFR T790M Mutation in Pericardial Effusion from a Non-Small Cell Lung Cancer Patient with Erlotinib Therapy.* Case Rep Oncol, 2013. **6**(1): p. 15-20.
27. Ogata, M., et al., *Spatial and temporal genetic heterogeneity of epidermal growth factor receptor gene status in a patient with non-small cell lung cancer: a case report.* J Med Case Rep, 2011. **5**: p. 553.
28. Scher, K.S., et al., *EGFR-mutated lung cancer with T790M-acquired resistance in the brain and histologic transformation in the lung.* J Natl Compr Canc Netw, 2013. **11**(9): p. 1040-4.
29. Li, S., et al., *Response to pemetrexed rechallenge after acquired resistance of EGFR-TKI in a patient with advanced NSCLC.* Lung Cancer, 2014. **84**(2): p. 203-5.
30. Bordi, P., et al., *Overcoming T790M-driven acquired resistance to EGFR-TKIs in NSCLC with afatinib: a case report.* Tumori, 2014. **100**(1): p. e20-3.
31. Phelps, M.A., et al., *Erlotinib in African Americans with advanced non-small cell lung cancer: a prospective randomized study with genetic and pharmacokinetic analyses.* Clin Pharmacol Ther, 2014. **96**(2): p. 182-91.
32. Furugen, M., et al., *An Autopsy Case of Two Distinct, Acquired Drug Resistance Mechanisms in Epidermal Growth Factor Receptor-mutant Lung Adenocarcinoma: Small Cell Carcinoma Transformation and Epidermal Growth Factor Receptor T790M Mutation.* Intern Med, 2015. **54**(19): p. 2491-6.
33. Belchis, D.A., et al., *Heterogeneity of resistance mutations detectable by nextgeneration sequencing in TKI-treated lung adenocarcinoma.* Oncotarget, 2016. **7**(29): p. 45237-45248.
34. Fujita, K., et al., *Concomitant T790M mutation and small-cell lung cancer transformation after acquired resistance to epidermal growth factor receptor-tyrosine kinase inhibitor.* Respirol Case Rep, 2017. **5**(1): p. e00206.
35. York, E.R., et al., *Tolerable and Effective Combination of Full-Dose Crizotinib and Osimertinib Targeting MET Amplification Sequentially Emerging after T790M Positivity in EGFR-Mutant Non-Small Cell Lung Cancer.* J Thorac Oncol, 2017. **12**(7): p. e85-e88.
36. Ruppert, A.M., et al., *EGFR-TKI and lung adenocarcinoma with CNS relapse: interest of molecular follow-up.* Eur Respir J, 2009. **33**(2): p. 436-40.
37. Clarke, J.L., et al., *High dose weekly erlotinib achieves therapeutic concentrations in CSF and is effective in leptomeningeal metastases from epidermal growth factor receptor mutant lung cancer.* J Neurooncol, 2010. **99**(2): p. 283-6.
38. Nakamura, T., et al., *A noninvasive system for monitoring resistance to epidermal growth factor receptor tyrosine kinase inhibitors with plasma DNA.* J Thorac Oncol, 2011. **6**(10): p. 1639-48.
39. van Riel, S., et al., *A patient with simultaneously appearing adenocarcinoma and small-cell lung carcinoma harbouring an identical EGFR exon 19 mutation.* Ann Oncol, 2012. **23**(12): p. 3188-9.
40. Rieux, C., et al., *[Biological diagnosis of resistance to erlotinib in a malignant pleural effusion].* Rev Mal Respir, 2013. **30**(7): p. 572-5.
41. Lozano, M.D., et al., *Variations in molecular profile in NSCLC can be analyzed using cytological samples: development of EGFR resistance mutations and coexistence of ALK-EML4 translocation in an EGFR-sensitive patient.* Int J Surg Pathol, 2015. **23**(2): p. 111-5.
42. Ou, S.I., et al., *Emergence of novel and dominant acquired EGFR solvent-front mutations at Gly796 (G796S/R) together with C797S/R and L792F/H mutations in one EGFR (L858R/T790M) NSCLC patient who progressed on osimertinib.* Lung Cancer, 2017. **108**: p. 228-231.
43. Helfrich, B.A., et al., *Antitumor activity of the epidermal growth factor receptor (EGFR) tyrosine kinase inhibitor gefitinib (ZD1839, Iressa) in non-small cell lung cancer cell lines correlates with gene copy number and EGFR mutations but not EGFR protein levels.* Clin Cancer Res, 2006. **12**(23): p. 7117-25.

44. Okabe, T., et al., *Differential constitutive activation of the epidermal growth factor receptor in non-small cell lung cancer cells bearing EGFR gene mutation and amplification.* Cancer Res, 2007. **67**(5): p. 2046-53.
45. Costa, D.B., et al., *BIM mediates EGFR tyrosine kinase inhibitor-induced apoptosis in lung cancers with oncogenic EGFR mutations.* PLoS Med, 2007. **4**(10): p. 1669-79; discussion 1680.
46. Yang, C.H., et al., *Specific EGFR mutations predict treatment outcome of stage IIIB/IV patients with chemotherapy-naive non-small-cell lung cancer receiving first-line gefitinib monotherapy.* J Clin Oncol, 2008. **26**(16): p. 2745-53.
47. Wu, S.G., et al., *Lung adenocarcinoma with good response to erlotinib after gefitinib treatment failure and acquired T790M mutation.* J Thorac Oncol, 2008. **3**(4): p. 451-2.
48. Wu, J.Y., et al., *Lung cancer with epidermal growth factor receptor exon 20 mutations is associated with poor gefitinib treatment response.* Clin Cancer Res, 2008. **14**(15): p. 4877-82.
49. Sugio, K., et al., *Prospective phase II study of gefitinib in non-small cell lung cancer with epidermal growth factor receptor gene mutations.* Lung Cancer, 2009. **64**(3): p. 314-8.
50. Ushiki, A., et al., *Genetic heterogeneity of EGFR mutation in pleomorphic carcinoma of the lung: response to gefitinib and clinical outcome.* Jpn J Clin Oncol, 2009. **39**(4): p. 267-70.
51. Kalikaki, A., et al., *Comparison of EGFR and K-RAS gene status between primary tumours and corresponding metastases in NSCLC.* Br J Cancer, 2008. **99**(6): p. 923-9.
52. Donovan, M.J., et al., *A systems pathology model for predicting overall survival in patients with refractory, advanced non-small-cell lung cancer treated with gefitinib.* Eur J Cancer, 2009. **45**(8): p. 1518-26.
53. Katayama, T., et al., *Efficacy of erlotinib for brain and leptomeningeal metastases in patients with lung adenocarcinoma who showed initial good response to gefitinib.* J Thorac Oncol, 2009. **4**(11): p. 1415-9.
54. Uramoto, H., et al., *Epithelial-mesenchymal transition in EGFR-TKI acquired resistant lung adenocarcinoma.* Anticancer Res, 2010. **30**(7): p. 2513-7.
55. Ready, N., et al., *Chemoradiotherapy and gefitinib in stage III non-small cell lung cancer with epidermal growth factor receptor and KRAS mutation analysis: cancer and leukemia group B (CALEB) 30106, a CALGB-stratified phase II trial.* J Thorac Oncol, 2010. **5**(9): p. 1382-90.
56. Price, K.A., et al., *Phase II trial of gefitinib and everolimus in advanced non-small cell lung cancer.* J Thorac Oncol, 2010. **5**(10): p. 1623-9.
57. Heon, S., et al., *Development of central nervous system metastases in patients with advanced non-small cell lung cancer and somatic EGFR mutations treated with gefitinib or erlotinib.* Clin Cancer Res, 2010. **16**(23): p. 5873-82.
58. Suda, K., et al., *Reciprocal and complementary role of MET amplification and EGFR T790M mutation in acquired resistance to kinase inhibitors in lung cancer.* Clin Cancer Res, 2010. **16**(22): p. 5489-98.
59. Uramoto, H., et al., *Expression of selected gene for acquired drug resistance to EGFR-TKI in lung adenocarcinoma.* Lung Cancer, 2011. **73**(3): p. 361-5.
60. Hata, A., et al., *Do complex mutations of the epidermal growth factor receptor gene reflect intratumoral heterogeneity?* J Thorac Oncol, 2011. **6**(6): p. 1144-6.
61. Yano, S., et al., *Hepatocyte growth factor expression in EGFR mutant lung cancer with intrinsic and acquired resistance to tyrosine kinase inhibitors in a Japanese cohort.* J Thorac Oncol, 2011. **6**(12): p. 2011-7.
62. Su, K.Y., et al., *Pretreatment epidermal growth factor receptor (EGFR) T790M mutation predicts shorter EGFR tyrosine kinase inhibitor response duration in patients with non-small-cell lung cancer.* J Clin Oncol, 2012. **30**(4): p. 433-40.
63. Tabara, K., et al., *Loss of activating EGFR mutant gene contributes to acquired resistance to EGFR tyrosine kinase inhibitors in lung cancer cells.* PLoS One, 2012. **7**(7): p. e41017.
64. Uramoto, H., et al., *Prognostic value of acquired resistance-related molecules in Japanese patients with NSCLC treated with an EGFR-TKI.* Anticancer Res, 2012. **32**(9): p. 3785-90.
65. Iwanaga, K., et al., *The long-term survival of a patient with adenosquamous lung carcinoma harboring EGFR-activating mutations who was treated with gefitinib.* Intern Med, 2012. **51**(19): p. 2771-4.
66. Sato, K., et al., *CBDCA + Pemetrexed + Bevacizumab and Its Maintenance Chemotherapy in a Case of Solitary Breast Metastasis from a Lung Adenocarcinoma Resistant to Gefitinib.* Case Rep Oncol, 2012. **5**(3): p. 546-53.
67. Hata, A., et al., *Does T790M disappear? Successful gefitinib rechallenge after T790M disappearance in a patient with EGFR-mutant non-small-cell lung cancer.* J Thorac Oncol, 2013. **8**(3): p. e27-9.
68. Kim, E.Y., et al., *Repeated favorable responses to epidermal growth factor receptor-tyrosine kinase inhibitors in a case of advanced lung adenocarcinoma.* Tuberc Respir Dis (Seoul), 2013. **74**(3): p. 129-33.
69. Keam, B., et al., *Rare and complex mutations of epidermal growth factor receptor, and efficacy of tyrosine kinase inhibitor in patients with non-small cell lung cancer.* Int J Clin Oncol, 2014. **19**(4): p. 594-600.

70. Ji, W., et al., *Mechanisms of acquired resistance to EGFR-tyrosine kinase inhibitor in Korean patients with lung cancer.* BMC Cancer, 2013. **13**: p. 606.
71. Kim, H.R., et al., *Prediction for response duration to epidermal growth factor receptor-tyrosine kinase inhibitors in EGFR mutated never smoker lung adenocarcinoma.* Lung Cancer, 2014. **83**(3): p. 374-82.
72. Ohara, S., et al., *Brain metastasis effectively treated with erlotinib following the acquisition of resistance to gefitinib: a case report.* J Med Case Rep, 2014. **8**: p. 64.
73. Li, H., et al., *Primary concomitant EGFR T790M mutation predicted worse prognosis in non-small cell lung cancer patients.* Onco Targets Ther, 2014. **7**: p. 513-24.
74. Wang, Y.F., et al., *Lung adenocarcinoma harboring L858R and T790M mutations in epidermal growth factor receptor, with poor response to gefitinib: A case report.* Oncol Lett, 2014. **8**(3): p. 1039-1042.
75. Suda, K., et al., *Small cell lung cancer transformation and T790M mutation: complimentary roles in acquired resistance to kinase inhibitors in lung cancer.* Sci Rep, 2015. **5**: p. 14447.
76. Vallee, A., et al., *Rapid clearance of circulating tumor DNA during treatment with AZD9291 of a lung cancer patient presenting the resistance EGFR T790M mutation.* Lung Cancer, 2016. **91**: p. 73-4.
77. Jukna, A., et al., *Squamous Cell Carcinoma "Transformation" Concurrent with Secondary T790M Mutation in Resistant EGFR-Mutated Adenocarcinomas.* J Thorac Oncol, 2016. **11**(4): p. e49-51.
78. Li, A., et al., *Acquired MET Y1248H and D1246N Mutations Mediate Resistance to MET Inhibitors in Non-Small Cell Lung Cancer.* Clin Cancer Res, 2017. **23**(16): p. 4929-4937.
79. Zheng, D., et al., *EGFR G796D mutation mediates resistance to osimertinib.* Oncotarget, 2017. **8**(30): p. 49671-49679.
80. Kang, J., et al., *Osimertinib and Cabozantinib Combinatorial Therapy in an EGFR-Mutant Lung Adenocarcinoma Patient with Multiple MET Secondary-Site Mutations after Resistance to Crizotinib.* J Thorac Oncol, 2018. **13**(4): p. e49-e53.
81. Kobayashi, S., et al., *EGFR mutation and resistance of non-small-cell lung cancer to gefitinib.* N Engl J Med, 2005. **352**(8): p. 786-92.
82. Shih, J.Y., C.H. Gow, and P.C. Yang, *EGFR mutation conferring primary resistance to gefitinib in non-small-cell lung cancer.* N Engl J Med, 2005. **353**(2): p. 207-8.
83. Uramoto, H., et al., *Epidermal growth factor receptor mutations are associated with gefitinib sensitivity in non-small cell lung cancer in Japanese.* Lung Cancer, 2006. **51**(1): p. 71-7.
84. Jackman, D.M., et al., *Response and resistance in a non-small-cell lung cancer patient with an epidermal growth factor receptor mutation and leptomeningeal metastases treated with high-dose gefitinib.* J Clin Oncol, 2006. **24**(27): p. 4517-20.
85. Kosaka, T., et al., *Analysis of epidermal growth factor receptor gene mutation in patients with non-small cell lung cancer and acquired resistance to gefitinib.* Clin Cancer Res, 2006. **12**(19): p. 5764-9.
86. Uramoto, H., et al., *Resistance to gefitinib.* Int J Clin Oncol, 2006. **11**(6): p. 487-91.
87. Ichihara, S., et al., *The impact of epidermal growth factor receptor gene status on gefitinib-treated Japanese patients with non-small-cell lung cancer.* Int J Cancer, 2007. **120**(6): p. 1239-47.
88. Soh, J., et al., *EGFR mutation status in pleural fluid predicts tumor responsiveness and resistance to gefitinib.* Lung Cancer, 2007. **56**(3): p. 445-8.
89. Ogino, A., et al., *Emergence of epidermal growth factor receptor T790M mutation during chronic exposure to gefitinib in a non small cell lung cancer cell line.* Cancer Res, 2007. **67**(16): p. 7807-14.
90. Miyazawa, H., et al., *Peptide nucleic acid-locked nucleic acid polymerase chain reaction clamp-based detection test for gefitinib-refractory T790M epidermal growth factor receptor mutation.* Cancer Sci, 2008. **99**(3): p. 595-600.
91. Chen, H.J., et al., *Clinicopathologic and molecular features of epidermal growth factor receptor T790M mutation and c-MET amplification in tyrosine kinase inhibitor-resistant Chinese non-small cell lung cancer.* Pathol Oncol Res, 2009. **15**(4): p. 651-8.
92. Onitsuka, T., et al., *Acquired resistance to gefitinib: the contribution of mechanisms other than the T790M, MET, and HGF status.* Lung Cancer, 2010. **68**(2): p. 198-203.
93. Kubo, T., et al., *Efficacy of a lumbo-peritoneal shunt for meningeal carcinomatosis refractory to gefitinib treatment.* Anticancer Res, 2009. **29**(7): p. 2759-60.
94. Zhao, J., et al., *Restriction endonuclease-mediated real-time digestion-PCR for somatic mutation detection.* Int J Cancer, 2013. **132**(12): p. 2858-66.
95. Graziano, P., et al., *EGFR-Driven Behavior and Intrapatient T790M Mutation Heterogeneity of Non-Small-Cell Carcinoma With Squamous Histology.* J Clin Oncol, 2015. **33**(31): p. e115-8.

96. Peng, L., Z.G. Song, and S.C. Jiao, *Efficacy analysis of tyrosine kinase inhibitors on rare non-small cell lung cancer patients harboring complex EGFR mutations.* Sci Rep, 2014. **4**: p. 6104.
97. Bersanelli, M., et al., *L718Q Mutation as New Mechanism of Acquired Resistance to AZD9291 in EGFR-Mutated NSCLC.* J Thorac Oncol, 2016. **11**(10): p. e121-3.
98. Song, H.N., et al., *Acquired C797S Mutation upon Treatment with a T790M-Specific Third-Generation EGFR Inhibitor (HM61713) in Non-Small Cell Lung Cancer.* J Thorac Oncol, 2016. **11**(4): p. e45-7.
99. Thress, K.S., et al., *Acquired EGFR C797S mutation mediates resistance to AZD9291 in non-small cell lung cancer harboring EGFR T790M.* Nat Med, 2015. **21**(6): p. 560-2.
100. Huang, J., et al., *Non-small cell lung cancer harboring a rare EGFR L747P mutation showing intrinsic resistance to both gefitinib and osimertinib (AZD9291): A case report.* Thorac Cancer, 2018. **9**(6): p. 745-749.
101. Oxnard, G.R., et al., *Acquired resistance to EGFR tyrosine kinase inhibitors in EGFR-mutant lung cancer: distinct natural history of patients with tumors harboring the T790M mutation.* Clin Cancer Res, 2011. **17**(6): p. 1616-22.
102. Ohashi, K., et al., *Lung cancers with acquired resistance to EGFR inhibitors occasionally harbor BRAF gene mutations but lack mutations in KRAS, NRAS, or MEK1.* Proc Natl Acad Sci U S A, 2012. **109**(31): p. E2127-33.
103. Shien, K., et al., *Acquired resistance to EGFR inhibitors is associated with a manifestation of stem cell-like properties in cancer cells.* Cancer Res, 2013. **73**(10): p. 3051-61.
104. Sun, J.M., et al., *Clinical implications of T790M mutation in patients with acquired resistance to EGFR tyrosine kinase inhibitors.* Lung Cancer, 2013. **82**(2): p. 294-8.
105. Hata, A., et al., *Rebiopsy of non-small cell lung cancer patients with acquired resistance to epidermal growth factor receptor-tyrosine kinase inhibitor: Comparison between T790M mutation-positive and mutation-negative populations.* Cancer, 2013. **119**(24): p. 4325-32.
106. Jin, Y., et al., *Mutational profiling of non-small-cell lung cancer patients resistant to first-generation EGFR tyrosine kinase inhibitors using next generation sequencing.* Oncotarget, 2016. **7**(38): p. 61755-61763.
107. Pietanza, M.C., et al., *Phase II study of the multitargeted tyrosine kinase inhibitor XL647 in patients with non-small-cell lung cancer.* J Thorac Oncol, 2012. **7**(5): p. 856-65.
108. Bahcall, M., et al., *Acquired METD1228V Mutation and Resistance to MET Inhibition in Lung Cancer.* Cancer Discov, 2016. **6**(12): p. 1334-1341.
109. Heist, R.S., et al., *Acquired Resistance to Crizotinib in NSCLC with MET Exon 14 Skipping.* J Thorac Oncol, 2016. **11**(8): p. 1242-1245.
110. Ou, S.-H.I., et al., *Emergence of Preexisting MET Y1230C Mutation as a Resistance Mechanism to Crizotinib in NSCLC with MET Exon 14 Skipping.* Journal of thoracic oncology : official publication of the International Association for the Study of Lung Cancer, 2017. **12**(1): p. 137-140.
111. Dong, H.J., et al., *Response and acquired resistance to crizotinib in Chinese patients with lung adenocarcinomas harboring MET Exon 14 splicing alternations.* Lung Cancer, 2016. **102**: p. 118-121.
112. Lu, X., et al., *MET Exon 14 Mutation Encodes an Actionable Therapeutic Target in Lung Adenocarcinoma.* Cancer Res, 2017. **77**(16): p. 4498-4505.
113. Yoshimura, K., et al., *Successful crizotinib monotherapy in EGFR-mutant lung adenocarcinoma with acquired MET amplification after erlotinib therapy.* Respir Med Case Rep, 2017. **20**: p. 160-163.
114. Dziadziuszko, R., et al., *An Activating KIT Mutation Induces Crizotinib Resistance in ROS1-Positive Lung Cancer.* J Thorac Oncol, 2016. **11**(8): p. 1273-1281.
115. Quintas-Cardama, A., et al., *Outcome of patients with chronic myeloid leukemia with multiple ABL1 kinase domain mutations receiving tyrosine kinase inhibitor therapy.* Haematologica, 2011. **96**(6): p. 918-21.
116. Redaelli, S., et al., *Three novel patient-derived BCR/ABL mutants show different sensitivity to second and third generation tyrosine kinase inhibitors.* American journal of hematology, 2012. **87**(11): p. E125-8.
117. Jabbour, E., et al., *Characteristics and outcomes of patients with V299L BCR-ABL kinase domain mutation after therapy with tyrosine kinase inhibitors.* Blood, 2012. **120**(16): p. 3382-3.
118. Breccia, M., et al., *Sequential development of mutant clones in an imatinib resistant chronic myeloid leukaemia patient following sequential treatment with multiple tyrosine kinase inhibitors: an emerging problem?* Cancer Chemother Pharmacol, 2009. **64**(1): p. 195-7.
119. Jabbour, E., et al., *Results of allogeneic hematopoietic stem cell transplantation for chronic myelogenous leukemia patients who failed tyrosine kinase inhibitors after developing BCR-ABL1 kinase domain mutations.* Blood, 2011. **117**(13): p. 3641-7.

120. Sakai, K., et al., *A novel insertion mutation of K294RGG within BCR-ABL kinase domain confers imatinib resistance: sequential analysis of the clonal evolution in a patient with chronic myeloid leukemia in blast crisis.* Int J Hematol, 2011. **93**(2): p. 237-242.
121. Foa, R., et al., *Dasatinib as first-line treatment for adult patients with Philadelphia chromosome-positive acute lymphoblastic leukemia.* Blood, 2011. **118**(25): p. 6521-8.
122. Razga, F., et al., *Role of treatment in the appearance and selection of BCR-ABL1 kinase domain mutations.* Mol Diagn Ther, 2012. **16**(4): p. 251-9.
123. Ravandi, F., et al., *Long-term follow-up of a phase 2 study of chemotherapy plus dasatinib for the initial treatment of patients with Philadelphia chromosome-positive acute lymphoblastic leukemia.* Cancer, 2015. **121**(23): p. 4158-64.
124. Branford, S., et al., *High frequency of point mutations clustered within the adenosine triphosphate-binding region of BCR/ABL in patients with chronic myeloid leukemia or Ph-positive acute lymphoblastic leukemia who develop imatinib (STI571) resistance.* Blood, 2002. **99**(9): p. 3472-5.
125. Shah, N.P., et al., *Multiple BCR-ABL kinase domain mutations confer polyclonal resistance to the tyrosine kinase inhibitor imatinib (STI571) in chronic phase and blast crisis chronic myeloid leukemia.* Cancer Cell, 2002. **2**(2): p. 117-25.
126. Hochhaus, A., et al., *Molecular and chromosomal mechanisms of resistance to imatinib (STI571) therapy.* Leukemia, 2002. **16**(11): p. 2190-6.
127. Branford, S., et al., *Detection of BCR-ABL mutations in patients with CML treated with imatinib is virtually always accompanied by clinical resistance, and mutations in the ATP phosphate-binding loop (P-loop) are associated with a poor prognosis.* Blood, 2003. **102**(1): p. 276-83.
128. Al-Ali, H.K., et al., *High incidence of BCR-ABL kinase domain mutations and absence of mutations of the PDGFR and KIT activation loops in CML patients with secondary resistance to imatinib.* Hematol J, 2004. **5**(1): p. 55-60.
129. Sorel, N., et al., *Evidence of ABL-kinase domain mutations in highly purified primitive stem cell populations of patients with chronic myelogenous leukemia.* Biochem Biophys Res Commun, 2004. **323**(3): p. 728-30.
130. Gruber, F.X., et al., *A novel Bcr-Abl splice isoform is associated with the L248V mutation in CML patients with acquired resistance to imatinib.* Leukemia, 2006. **20**(11): p. 2057-60.
131. Jabbour, E., et al., *Long-term outcome of patients with chronic myeloid leukemia treated with second-generation tyrosine kinase inhibitors after imatinib failure is predicted by the in vitro sensitivity of BCR-ABL kinase domain mutations.* Blood, 2009. **114**(10): p. 2037-43.
132. Press, R.D., et al., *Determining the rise in BCR-ABL RNA that optimally predicts a kinase domain mutation in patients with chronic myeloid leukemia on imatinib.* Blood, 2009. **114**(13): p. 2598-605.
133. Kim, W.S., et al., *Dynamic change of T315I BCR-ABL kinase domain mutation in Korean chronic myeloid leukaemia patients during treatment with Abl tyrosine kinase inhibitors.* Hematol Oncol, 2010. **28**(2): p. 82-8.
134. Markose, P., et al., *Spectrum of BCR-ABL kinase domain mutations in patients with chronic myeloid leukemia from India with suspected resistance to imatinib-mutations are rare and have different distributions.* Leuk Lymphoma, 2009. **50**(12): p. 2092-5.
135. Rajappa, S., et al., *Kinase domain mutations and responses to dose escalation in chronic myeloid leukemia resistant to standard dose imatinib mesylate.* Leuk Lymphoma, 2010. **51**(1): p. 79-84.
136. Sharma, P., et al., *Mutations in ABL kinase domain are associated with inferior progression-free survival.* Leuk Lymphoma, 2010. **51**(6): p. 1072-8.
137. Manrique Arechavaleta, G., et al., *Rapid and sensitive allele-specific (AS)-RT-PCR assay for detection of T315I mutation in chronic myeloid leukemia patients treated with tyrosine-kinase inhibitors.* Clin Exp Med, 2011. **11**(1): p. 55-9.
138. Martin, S.E., et al., *Chronic myeloid leukemia with e19a2 atypical transcript: early imatinib resistance and complete response to dasatinib.* Cancer Genet Cytogenet, 2010. **201**(2): p. 133-4.
139. Qin, Y., et al., *Characteristics of BCR-ABL kinase domain point mutations in Chinese imatinib-resistant chronic myeloid leukemia patients.* Ann Hematol, 2011. **90**(1): p. 47-52.
140. Gruber, F.X., et al., *BCR-ABL isoforms associated with intrinsic or acquired resistance to imatinib: more heterogeneous than just ABL kinase domain point mutations?* Med Oncol, 2012. **29**(1): p. 219-26.
141. Ono, T., et al., *BCR-ABL1 mutations in patients with imatinib-resistant Philadelphia chromosome-positive leukemia by use of the PCR-Invader assay.* Leuk Res, 2011. **35**(5): p. 598-603.
142. Bengio, R.M., et al., *Clinical outcome of chronic myeloid leukemia imatinib-resistant patients: do BCR-ABL kinase domain mutations affect patient survival? First multicenter Argentinean study.* Leuk Lymphoma, 2011. **52**(9): p. 1720-6.

143. Chang, B.H., et al., *Imatinib resistant BCR-ABL1 mutations at relapse in children with Ph+ ALL: a Children's Oncology Group (COG) study.* Br J Haematol, 2012. **157**(4): p. 507-10.
144. Razga, F., et al., *Analysis of mutations in the BCR-ABL1 kinase domain, using direct sequencing: detection of the T315I mutation in bone marrow CD34+ cells of a patient with chronic myelogenous leukemia 6 months prior to its emergence in peripheral blood.* Mol Diagn Ther, 2012. **16**(3): p. 163-6.
145. Tiribelli, M., et al., *Impact of BCR-ABL mutations on response to dasatinib after imatinib failure in elderly patients with chronic-phase chronic myeloid leukemia.* Ann Hematol, 2013. **92**(2): p. 179-83.
146. Elias, M.H., et al., *Contribution of BCR-ABL kinase domain mutations to imatinib mesylate resistance in Philadelphia chromosome positive Malaysian chronic myeloid leukemia patients.* Hematol Rep, 2012. **4**(4): p. e23.
147. Marce, S., et al., *Frequency of ABL gene mutations in chronic myeloid leukemia patients resistant to imatinib and results of treatment switch to second-generation tyrosine kinase inhibitors.* Med Clin (Barc), 2013. **141**(3): p. 95-9.
148. Chahardouli, B., et al., *Detection of BCR-ABL kinase domain mutations in patients with chronic myeloid leukemia on imatinib.* Hematology, 2013. **18**(6): p. 328-33.
149. Parker, W.T., et al., *BCR-ABL1 kinase domain mutations may persist at very low levels for many years and lead to subsequent TKI resistance.* Br J Cancer, 2013. **109**(6): p. 1593-8.
150. Elias, M.H., et al., *BCR-ABL kinase domain mutations, including 2 novel mutations in imatinib resistant Malaysian chronic myeloid leukemia patients-Frequency and clinical outcome.* Leuk Res, 2014. **38**(4): p. 454-9.
151. Rostami, G., et al., *Incidence and clinical importance of BCR-ABL1 mutations in Iranian patients with chronic myeloid leukemia on imatinib.* J Hum Genet, 2015. **60**(5): p. 253-8.
152. Li, C., et al., *E255K and G250E mutation appearing in a patient with e19a2 chronic myeloid leukemia resistant to imatinib.* Clin Lab, 2015. **61**(1-2): p. 183-6.
153. Rejali, L., et al., *Characterizing of Four Common BCR-ABL Kinase Domain Mutations (T315I, Y253H, M351T and E255K) in Iranian Chronic Myelogenous Leukemia Patients With Imatinib Resistance.* Iran J Cancer Prev, 2015. **8**(3): p. e2334.
154. Verma, D., et al., *Complexity of BCR-ABL kinase domain mutations during the course of therapy with tyrosine kinase inhibitors in chronic myeloid leukemia.* Am J Hematol, 2009. **84**(4): p. 256-7.
155. Hayette, S., et al., *Longitudinal studies of SRC family kinases in imatinib- and dasatinib-resistant chronic myelogenous leukemia patients.* Leuk Res, 2011. **35**(1): p. 38-43.
156. Strhakova, L., et al., *Use of direct sequencing for detection of mutations in the BCR-ABL kinase domain in Slovak patients with chronic myeloid leukemia.* Neoplasma, 2011. **58**(6): p. 548-53.
157. Sacha, T., et al., *[ABL domain kinase point mutations as a cause of resistance to therapy of patients with chronic myeloid leukemia with tyrosine kinase inhibitors. Single center experience].* Przegl Lek, 2011. **68**(5): p. 253-7.
158. Ferri, C., et al., *Early detection and quantification of mutations in the tyrosine kinase domain of chimerical BCR-ABL1 gene combining high-resolution melting analysis and mutant-allele specific quantitative polymerase chain reaction.* Leuk Lymphoma, 2013. **54**(3): p. 598-606.
159. Cortes, J.E., et al., *Ponatinib in refractory Philadelphia chromosome-positive leukemias.* N Engl J Med, 2012. **367**(22): p. 2075-88.
160. Khorashad, J.S., et al., *BCR-ABL1 compound mutations in tyrosine kinase inhibitor-resistant CML: frequency and clonal relationships.* Blood, 2013. **121**(3): p. 489-98.
161. Furman, R.R., et al., *Ibrutinib resistance in chronic lymphocytic leukemia.* N Engl J Med, 2014. **370**(24): p. 2352-4.
162. Woyach, J.A., et al., *Resistance mechanisms for the Bruton's tyrosine kinase inhibitor ibrutinib.* N Engl J Med, 2014. **370**(24): p. 2286-94.
163. Chiron, D., et al., *Cell-cycle reprogramming for PI3K inhibition overrides a relapse-specific C481S BTK mutation revealed by longitudinal functional genomics in mantle cell lymphoma.* Cancer Discov, 2014. **4**(9): p. 1022-35.
164. Maddocks, K.J., et al., *Etiology of Ibrutinib Therapy Discontinuation and Outcomes in Patients With Chronic Lymphocytic Leukemia.* JAMA Oncol, 2015. **1**(1): p. 80-7.
165. Burger, J.A., et al., *Clonal evolution in patients with chronic lymphocytic leukaemia developing resistance to BTK inhibition.* Nat Commun, 2016. **7**: p. 11589.
166. Sharma, S., et al., *Identification of a structurally novel BTK mutation that drives ibrutinib resistance in CLL.* Oncotarget, 2016. **7**(42): p. 68833-68841.
167. Ahn, I.E., et al., *Clonal evolution leading to ibrutinib resistance in chronic lymphocytic leukemia.* Blood, 2017. **129**(11): p. 1469-1479.

168. Moore, A.S., et al., *Selective FLT3 inhibition of FLT3-ITD+ acute myeloid leukaemia resulting in secondary D835Y mutation: a model for emerging clinical resistance patterns.* Leukemia, 2012. **26**(7): p. 1462-70.
169. Smith, C.C., et al., *Validation of ITD mutations in FLT3 as a therapeutic target in human acute myeloid leukaemia.* Nature, 2012. **485**(7397): p. 260-3.
170. Albers, C., et al., *The secondary FLT3-ITD F691L mutation induces resistance to AC220 in FLT3-ITD+ AML but retains in vitro sensitivity to PKC412 and Sunitinib.* Leukemia, 2013. **27**(6): p. 1416-8.
171. Man, C.H., et al., *Sorafenib treatment of FLT3-ITD(+) acute myeloid leukemia: favorable initial outcome and mechanisms of subsequent nonresponsiveness associated with the emergence of a D835 mutation.* Blood, 2012. **119**(22): p. 5133-43.
172. Baker, S.D., et al., *Emergence of polyclonal FLT3 tyrosine kinase domain mutations during sequential therapy with sorafenib and sunitinib in FLT3-ITD-positive acute myeloid leukemia.* Clin Cancer Res, 2013. **19**(20): p. 5758-68.
173. Zheng, S., et al., *KIT and BRAF heterogeneous mutations in gastrointestinal stromal tumors after secondary imatinib resistance.* Gastric Cancer, 2015. **18**(4): p. 796-802.
174. Debiec-Rychter, M., et al., *Mechanisms of resistance to imatinib mesylate in gastrointestinal stromal tumors and activity of the PKC412 inhibitor against imatinib-resistant mutants.* Gastroenterology, 2005. **128**(2): p. 270-9.
175. Antonescu, C.R., et al., *Acquired resistance to imatinib in gastrointestinal stromal tumor occurs through secondary gene mutation.* Clin Cancer Res, 2005. **11**(11): p. 4182-90.
176. Wardelmann, E., et al., *Polyclonal evolution of multiple secondary KIT mutations in gastrointestinal stromal tumors under treatment with imatinib mesylate.* Clin Cancer Res, 2006. **12**(6): p. 1743-9.
177. Heinrich, M.C., et al., *Molecular correlates of imatinib resistance in gastrointestinal stromal tumors.* J Clin Oncol, 2006. **24**(29): p. 4764-74.
178. Desai, J., et al., *Clonal evolution of resistance to imatinib in patients with metastatic gastrointestinal stromal tumors.* Clin Cancer Res, 2007. **13**(18 Pt 1): p. 5398-405.
179. Sym, S.J., et al., *Surgical intervention following imatinib treatment in patients with advanced gastrointestinal stromal tumors (GISTs).* J Surg Oncol, 2008. **98**(1): p. 27-33.
180. Chen, L.L., et al., *Evolution from heterozygous to homozygous KIT mutation in gastrointestinal stromal tumor correlates with the mechanism of mitotic nondisjunction and significant tumor progression.* Mod Pathol, 2008. **21**(7): p. 826-36.
181. Liegl, B., et al., *Heterogeneity of kinase inhibitor resistance mechanisms in GIST.* J Pathol, 2008. **216**(1): p. 64-74.
182. Liegl, B., et al., *Rhabdomyosarcomatous differentiation in gastrointestinal stromal tumors after tyrosine kinase inhibitor therapy: a novel form of tumor progression.* Am J Surg Pathol, 2009. **33**(2): p. 218-26.
183. Heinrich, M.C., et al., *Primary and secondary kinase genotypes correlate with the biological and clinical activity of sunitinib in imatinib-resistant gastrointestinal stromal tumor.* J Clin Oncol, 2008. **26**(33): p. 5352-9.
184. Bauer, S., et al., *Proapoptotic activity of bortezomib in gastrointestinal stromal tumor cells.* Cancer Res, 2010. **70**(1): p. 150-9.
185. Revheim, M.E., et al., *Establishment and characterization of a human gastrointestinal stromal tumour (GIST) xenograft in athymic nude mice.* Anticancer Res, 2009. **29**(11): p. 4331-6.
186. Wang, C.M., et al., *Molecular mechanisms of secondary imatinib resistance in patients with gastrointestinal stromal tumors.* J Cancer Res Clin Oncol, 2010. **136**(7): p. 1065-71.
187. Antonescu, C.R., et al., *Dedifferentiation in gastrointestinal stromal tumor to an anaplastic KIT-negative phenotype: a diagnostic pitfall: morphologic and molecular characterization of 8 cases occurring either de novo or after imatinib therapy.* Am J Surg Pathol, 2013. **37**(3): p. 385-92.
188. Gao, J., et al., *Secondary mutations of c-KIT contribute to acquired resistance to imatinib and decrease efficacy of sunitinib in Chinese patients with gastrointestinal stromal tumors.* Med Oncol, 2013. **30**(2): p. 522.
189. Kang, G., et al., *Detection of KIT and PDGFRA mutations in the plasma of patients with gastrointestinal stromal tumor.* Target Oncol, 2015. **10**(4): p. 597-601.
190. Heydt, C., et al., *Massively parallel sequencing fails to detect minor resistant subclones in tissue samples prior to tyrosine kinase inhibitor therapy.* BMC Cancer, 2015. **15**: p. 291.
191. Spitaleri, G., et al., *Inactivity of imatinib in gastrointestinal stromal tumors (GISTs) harboring a KIT activation-loop domain mutation (exon 17 mutation pN822K).* Onco Targets Ther, 2015. **8**: p. 1997-2003.
192. Wada, N., et al., *Detecting Secondary C-KIT Mutations in the Peripheral Blood of Patients with Imatinib-Resistant Gastrointestinal Stromal Tumor.* Oncology, 2016. **90**(2): p. 112-7.
193. Takahashi, T., et al., *Genomic and transcriptomic analysis of imatinib resistance in gastrointestinal stromal tumors.* Genes Chromosomes Cancer, 2017. **56**(4): p. 303-313.

194. Sugase, T., et al., *Surgical resection of recurrent gastrointestinal stromal tumor after interruption of long-term nilotinib therapy.* Surg Case Rep, 2016. **2**(1): p. 137.
195. Kikuchi, H., et al., *Surgical intervention for imatinib and sunitinib-resistant gastrointestinal stromal tumors.* Int J Clin Oncol, 2011. **16**(6): p. 741-5.
196. Hatzivassiliou, G., et al., *ERK inhibition overcomes acquired resistance to MEK inhibitors.* Mol Cancer Ther, 2012. **11**(5): p. 1143-54.
197. Hirota, S., et al., *Gain-of-function mutations of platelet-derived growth factor receptor alpha gene in gastrointestinal stromal tumors.* Gastroenterology, 2003. **125**(3): p. 660-7.
198. Heinrich, M.C., et al., *Kinase mutations and imatinib response in patients with metastatic gastrointestinal stromal tumor.* J Clin Oncol, 2003. **21**(23): p. 4342-9.
199. Xu, X., et al., *Gastrointestinal stromal tumor with structures resembling intracytoplasmic lumina.* Ultrastruct Pathol, 2010. **34**(5): p. 301-6.
200. Cassier, P.A., et al., *Outcome of patients with platelet-derived growth factor receptor alpha-mutated gastrointestinal stromal tumors in the tyrosine kinase inhibitor era.* Clin Cancer Res, 2012. **18**(16): p. 4458-64.
201. Yoo, C., et al., *Efficacy of Imatinib in Patients with Platelet-Derived Growth Factor Receptor Alpha-Mutated Gastrointestinal Stromal Tumors.* Cancer Res Treat, 2016. **48**(2): p. 546-52.
202. Farag, S., et al., *Clinical characteristics and treatment outcome in a large multicentre observational cohort of PDGFRA exon 18 mutated gastrointestinal stromal tumour patients.* Eur J Cancer, 2017. **76**: p. 76-83.
203. Rizos, H., et al., *BRAF inhibitor resistance mechanisms in metastatic melanoma: spectrum and clinical impact.* Clin Cancer Res, 2014. **20**(7): p. 1965-77.
204. Kwong, L.N., et al., *Co-clinical assessment identifies patterns of BRAF inhibitor resistance in melanoma.* The Journal of clinical investigation, 2015. **125**(4): p. 1459-70.
205. Hoogstraat, M., et al., *Detailed imaging and genetic analysis reveal a secondary BRAF(L505H) resistance mutation and extensive intrapatient heterogeneity in metastatic BRAF mutant melanoma patients treated with vemurafenib.* Pigment Cell Melanoma Res, 2015. **28**(3): p. 318-23.
206. Monsma, D.J., et al., *Melanoma patient derived xenografts acquire distinct Vemurafenib resistance mechanisms.* Am J Cancer Res, 2015. **5**(4): p. 1507-18.
207. Mologni, L., et al., *Concomitant BCORL1 and BRAF Mutations in Vemurafenib-Resistant Melanoma Cells.* Neoplasia, 2018. **20**(5): p. 467-477.
208. Cho, J., et al., *Emergence of CTNNB1 mutation at acquired resistance to KIT inhibitor in metastatic melanoma.* Clin Transl Oncol, 2017. **19**(10): p. 1247-1252.
209. Zaretsky, J.M., et al., *Mutations Associated with Acquired Resistance to PD-1 Blockade in Melanoma.* N Engl J Med, 2016. **375**(9): p. 819-29.
210. Trunzer, K., et al., *Pharmacodynamic effects and mechanisms of resistance to vemurafenib in patients with metastatic melanoma.* J Clin Oncol, 2013. **31**(14): p. 1767-74.
211. Long, G.V., et al., *Increased MAPK reactivation in early resistance to dabrafenib/trametinib combination therapy of BRAF-mutant metastatic melanoma.* Nature communications, 2014. **5**: p. 5694.
212. Rizos, H., et al., *BRAF inhibitor resistance mechanisms in metastatic melanoma: spectrum and clinical impact.* Clinical cancer research : an official journal of the American Association for Cancer Research, 2014. **20**(7): p. 1965-77.
213. Nazarian, R., et al., *Melanomas acquire resistance to B-RAF(V600E) inhibition by RTK or N-RAS upregulation.* Nature, 2010. **468**(7326): p. 973-7.
214. Brinkhuizen, T., et al., *Acquired resistance to the Hedgehog pathway inhibitor vismodegib due to smoothened mutations in treatment of locally advanced basal cell carcinoma.* J Am Acad Dermatol, 2014. **71**(5): p. 1005-8.
215. Pricl, S., et al., *Smoothened (SMO) receptor mutations dictate resistance to vismodegib in basal cell carcinoma.* Mol Oncol, 2015. **9**(2): p. 389-97.
216. Sharpe, H.J., et al., *Genomic analysis of smoothened inhibitor resistance in basal cell carcinoma.* Cancer Cell, 2015. **27**(3): p. 327-41.
217. Atwood, S.X., et al., *Smoothened variants explain the majority of drug resistance in basal cell carcinoma.* Cancer Cell, 2015. **27**(3): p. 342-53.
218. Li, S., et al., *Endocrine-therapy-resistant ESR1 variants revealed by genomic characterization of breast-cancer-derived xenografts.* Cell Rep, 2013. **4**(6): p. 1116-30.
219. Robinson, D.R., et al., *Activating ESR1 mutations in hormone-resistant metastatic breast cancer.* Nat Genet, 2013. **45**(12): p. 1446-51.

220. Toy, W., et al., *ESR1 ligand-binding domain mutations in hormone-resistant breast cancer.* Nat Genet, 2013. **45**(12): p. 1439-45.
221. Merenbakh-Lamin, K., et al., *D538G mutation in estrogen receptor-alpha: A novel mechanism for acquired endocrine resistance in breast cancer.* Cancer Res, 2013. **73**(23): p. 6856-64.
222. Guttery, D.S., et al., *Noninvasive detection of activating estrogen receptor 1 (ESR1) mutations in estrogen receptor-positive metastatic breast cancer.* Clin Chem, 2015. **61**(7): p. 974-82.
223. Takeshita, T., et al., *Droplet digital polymerase chain reaction assay for screening of ESR1 mutations in 325 breast cancer specimens.* Transl Res, 2015. **166**(6): p. 540-553 e2.
224. Niu, J., et al., *Incidence and clinical significance of ESR1 mutations in heavily pretreated metastatic breast cancer patients.* Onco Targets Ther, 2015. **8**: p. 3323-8.
225. Bardia, A., et al., *Metastatic Breast Cancer With ESR1 Mutation: Clinical Management Considerations From the Molecular and Precision Medicine (MAP) Tumor Board at Massachusetts General Hospital.* Oncologist, 2016. **21**(9): p. 1035-40.
226. Yanagawa, T., et al., *Detection of ESR1 mutations in plasma and tumors from metastatic breast cancer patients using next-generation sequencing.* Breast Cancer Res Treat, 2017. **163**(2): p. 231-240.
227. Rodrik-Outmezguine, V.S., et al., *Overcoming mTOR resistance mutations with a new-generation mTOR inhibitor.* Nature, 2016. **534**(7606): p. 272-6.
228. Chen, E.J., et al., *Abiraterone treatment in castration-resistant prostate cancer selects for progesterone responsive mutant androgen receptors.* Clin Cancer Res, 2015. **21**(6): p. 1273-80.
229. Romanel, A., et al., *Plasma AR and abiraterone-resistant prostate cancer.* Sci Transl Med, 2015. **7**(312): p. 312re10.
230. Culig, Z., et al., *Mutant androgen receptor detected in an advanced-stage prostatic carcinoma is activated by adrenal androgens and progesterone.* Mol Endocrinol, 1993. **7**(12): p. 1541-50.
231. Azad, A.A., et al., *Androgen Receptor Gene Aberrations in Circulating Cell-Free DNA: Biomarkers of Therapeutic Resistance in Castration-Resistant Prostate Cancer.* Clin Cancer Res, 2015. **21**(10): p. 2315-24.
232. Sperger, J.M., et al., *Integrated Analysis of Multiple Biomarkers from Circulating Tumor Cells Enabled by Exclusion-Based Analyte Isolation.* Clin Cancer Res, 2017. **23**(3): p. 746-756.
233. Taplin, M.E., et al., *Mutation of the androgen-receptor gene in metastatic androgen-independent prostate cancer.* N Engl J Med, 1995. **332**(21): p. 1393-8.
234. Taplin, M.E., et al., *Androgen receptor mutations in androgen-independent prostate cancer: Cancer and Leukemia Group B Study 9663.* J Clin Oncol, 2003. **21**(14): p. 2673-8.
235. Ceraline, J., et al., *Constitutive activation of the androgen receptor by a point mutation in the hinge region: a new mechanism for androgen-independent growth in prostate cancer.* Int J Cancer, 2004. **108**(1): p. 152-7.
236. Steinkamp, M.P., et al., *Treatment-dependent androgen receptor mutations in prostate cancer exploit multiple mechanisms to evade therapy.* Cancer Res, 2009. **69**(10): p. 4434-42.
237. Taplin, M.E., et al., *Selection for androgen receptor mutations in prostate cancers treated with androgen antagonist.* Cancer Res, 1999. **59**(11): p. 2511-5.
238. Diamond, J.R., et al., *Initial clinical sensitivity and acquired resistance to MET inhibition in MET-mutated papillary renal cell carcinoma.* J Clin Oncol, 2013. **31**(16): p. e254-8.
239. Wagle, N., et al., *Response and acquired resistance to everolimus in anaplastic thyroid cancer.* N Engl J Med, 2014. **371**(15): p. 1426-33.
240. Yauch, R.L., et al., *Smoothened mutation confers resistance to a Hedgehog pathway inhibitor in medulloblastoma.* Science, 2009. **326**(5952): p. 572-4.
241. Carlino, M.S., et al., *Antiproliferative effects of continued mitogen-activated protein kinase pathway inhibition following acquired resistance to BRAF and/or MEK inhibition in melanoma.* Molecular cancer therapeutics, 2013. **12**(7): p. 1332-42.
242. Wagle, N., et al., *Dissecting therapeutic resistance to RAF inhibition in melanoma by tumor genomic profiling.* J Clin Oncol, 2011. **29**(22): p. 3085-96.
243. Van Allen, E.M., et al., *The genetic landscape of clinical resistance to RAF inhibition in metastatic melanoma.* Cancer discovery, 2014. **4**(1): p. 94-109.
244. Wheler, J., et al., *Next generation sequencing of exceptional responders with BRAF-mutant melanoma: implications for sensitivity and resistance.* BMC Cancer, 2015. **15**: p. 61.
245. Gray, E.S., et al., *Circulating tumor DNA to monitor treatment response and detect acquired resistance in patients with metastatic melanoma.* Oncotarget, 2015. **6**(39): p. 42008-18.

© 2020 by the authors. Submitted for possible open access publication under the terms and conditions of the Creative Commons Attribution (CC BY) license (http://creativecommons.org/licenses/by/4.0/).
